# Characterization of phenotypic and genotypic traits of *Klebsiella pneumoniae* strains resistant to 3rd generation cephalosporins in hospital settings: A case study in Ho Chi Minh City, Vietnam

**DOI:** 10.1007/s11033-025-10493-4

**Published:** 2025-04-15

**Authors:** Minh Tuan Huynh, My Dung Jusselme, Hung Van Pham, Tien Dung Nguyen, Minh Quynh Chau

**Affiliations:** 1 Medical Microbiology Department, University Medical Center Ho Chi Minh City, 215 Hong Bang street, Ward 11, District 5, Ho Chi Minh City, Vietnam; 2 Department of Microbiology and Parasitology, University of Medicine and Pharmacy at Ho Chi Minh City, 217 Hong Bang street, Ward 11, District 5, Ho Chi Minh City, Vietnam; 3 LEESU, Univ Paris Est Creteil, ENPC, Institut Polytechnique de Paris, 61 avenue du Général de Gaulle, 94000 Creteil, France; 4 Nam Khoa Trading and Service Company Limited, 793/58 Tran Xuan Soan street, Tan Hung Ward, District 7, Ho Chi Minh City, Vietnam; 5 National Fisheries Inspection and Quanlity Assurance Branch 4, 271 To Ngoc Van street, Linh Dong Ward, Thu Duc City, Ho Chi Minh City, Vietnam

**Keywords:** *Klebsiella pneumoniae*, Phenotype trait, Genotype trait, AmpC β-lactamase, ESBL, Carbapenemase

## Abstract

**Background:**

*Klebsiella pneumoniae* is a dangerous pathogen, responsible for a variety of infections and has been growing resistance to antibiotics, making it difficult to treat diseases caused by this bacterium. A better understanding of the phenotypic and genotypic characteristics of this bacterium is essential for developing more effective treatments. This study examines the prevalence of *Klebsiella pneumoniae* in patient samples from eight hospitals in Ho Chi Minh City, Vietnam, in 2023, focusing on its AmpC β-lactamase, ESBL, and carbapenemase genotypes.

**Methods and Results:**

A total of 230 *Klebsiella pneumoniae* strains were isolated from clinical specimens, including sputum, pus, urine, bronchial lavage fluid, blood, and other fluids. The disk diffusion method detected the presence of these resistance enzymes, while multiplex real-time PCR identified the associated genes. Results showed that 78.7% of isolates produced AmpC β-lactamase, 21.3% produced ESBL, and 27.0% produced carbapenemase. ESBL-producing strains (90.9%) were more common than AmpC (67.4%) and carbapenemase (45.7%) strains. Additionally, 27.0% of isolates produced multiple resistance enzymes. The most prevalent AmpC gene was DHA (63.5%), while SHV (84.3%), TEM (55.2%), and CTX-M (70.0%) were the most common ESBL genes. OXA-48 (31.3%) was the dominant carbapenemase gene, followed by NDM1 (24.8%) and KPC (9.1%). On average, each strain carried 3.3 resistance genes.

**Conclusions:**

This study reveals a high prevalence of antimicrobial resistance in *Klebsiella pneumoniae* isolates from patient samples in Ho Chi Minh City hospitals, notably to third-generation cephalosporins and carbapenems. These findings highlight the need for ongoing surveillance and improved treatment strategies.

**Supplementary Information:**

The online version contains supplementary material available at 10.1007/s11033-025-10493-4.

## Introduction

*Klebsiella pneumoniae* (*K. pneumoniae*) is a Gram-negative enteric bacterium belonging to the family Enterobacteriaceae and infections caused by *K. pneumoniae* are particularly problematic due to rising antimicrobial resistance. *K. pneumoniae* is classified among the ESKAPE (*Enterococcus faecium, Staphylococcus aureus, Klebsiella pneumoniae, Acinetobacter baumannii, Pseudomonas aeruginosa*, and Enterobacter species) pathogens, a group of antimicrobial-resistance pathogens that play crucial roles in nosocomial infections, pathogenesis, and the dissemination of antibiotic resistance [1–3]. Of particuler concern is its resistance to cephalosporins, a versatile antibiotic group divised into 4 groups. First generation cephalosporins (*e.g.*, cefazolin, cephalothin) target Gram-positive bacteria, while second generation (*e.g.*, cefuroxime, cefamandole) offer increased activity against Gram-negative bacteria compared to the first generation. Third-generation cephalosporins (*e.g.*, cefotaxime, ceftazidime) have an even broader spectrum, and particularly against *Enterobacteriaceae*. Fourth generation cephalosporins (*e.g.* cefepime) provide a broader activity spectrum and effective against both Gram-positive and Gram-negative strains, including resistant Enterobacteriaceae and *Pseudomonas* [4]. This concern is heightened by the fact that *K. pneumoniae*, along with other ESKAPE pathogens, frequently develops multidrug-resistant (MDR) and extremely drug-resistant (XDR) strains, further limiting the effectiveness of available antibiotic treatments [5].

The acquisition and increas of antibiotic resistance in *K. pneumoniae* have led to a decline in the effectiveness of traditional treatments against this pathogen. The resistance to antibiotics in *K. pneumoniae* is mainly formed by five mechanisms: (1) production of enzymes (*e.i.*, extended-spectrum beta-lactamases (ESBLs) enzymes) that inactivate antibiotics, (2) alteration of antibiotic target structure, (3) loss or mutation of porins, reducing the permeability of the cell membrane, (4) increased activity of the pump system to push antibiotics out of the cell membrane, and (5) biofilm formation. Among these mechanismss, the production of antibiotic-inactivating enzymes is especially critical [6, 7]. *K. pneumoniae* secretes several types of β-lactamase enzymes, including AmpC β-lactamase, ESBLs and carbapenemase, which confer significant resistance. AmpC β-lactamase hydrolyzes most cephalosporins, cephamycins and monobactams. ESBLs can hydrolyse broad-spectrum cephalosporin and monobactam antibiotics, and are often resistant to fluoroquinolone and aminoglycoside antibiotics. Lastly, carbapenemases degrade several carbapenem antibiotics including ertapenem, imipenem, and meropenem, which are considered last-resort options for treatment [3].

Resistance to first- and second-line antimicrobial therapies has become widespread, with *K. pneumoniae* now recognised as a leading cause of neonatal sepsis in low- and middle-income countries, associated with case fatality rates of up to 30% [8, 9]. A systematic review involving 2462 patients infected with carbapenem-resistant *K. pneumoniae* reported an overall mortality of 42.14% [10]. Regional mortality rates for carbapenem-resistant strains are highest in Europe (50.06%), followed by South America (46.7%) and Asia (44.8%) [10]. In Seoul, South Korea, carbapenem-resistant *K. pneumoniae* accounted for 58.9% of isolates in a study spanning 2018–2020 [11]. In China, ESBL-producing *K. pneumoniae* showed a prevalence rate of 38.3%, with resistance rates of 40.0% to ceftazidime and 26.0% to cefotaxime [12]. In Vietnam, antibiotic resistance is an urgent issue exacerbated by unregulated antibiotic use. After China, Vietnam has the second-highest prevalence of *K. pneumoniae* producing extended spectrum β-lactamase, with 67% of urinary tract infection isolates affected [13]. In addition, the prevalence of carbapenemase-producing *K. pneumoniae* in Viet Nam is increasing dramatically, reaching 24% in 2016 [14]. In Ben Tre province, at Nguyen Dinh Chieu Hospital, the rates of *K. pneumoniae* resistance to 2nd and 3rd generation cephalosporins, with ESBL resistance of 33.3% and carbapenem resistance ranging from 25 to 30% were observed [15]. At Can Tho General Central Hospital, cephalosporin resistance reached 78.1% to 83.8%, with imipenem resistance at 54.5% in 2019, and multidrug resistance climbed to 89.1% by 2022, with ESBL enzyme production at 31.3% [16]. Furthermore, AmpC β-lactamase enzyme production was reported at 48.9% at in the same hospital [17].

The use of routine methods, such as culture and conventional biochemical tests, to isolate pathogenic strains of *K. pneumoniae* often yields inaccurate results, due to biochemical similarities between *K. pneumoniae* and other coliform bacteria [18]. The complexity of *K. pneumoniae* resistance is further compounded by its ability to carry multiple resistance genes or produce various resistance enzymes. In Ho Chi Minh City, at the University Medical Center, the situation of *K. pneumoniae* bacteria causing central venous infusion related bacteremia shows high rates of β-lactam resistance, with ceftazidime and ceftriaxone resistance reaching 70.0%, and cefoxitin resistance standing at 50.0% [19]. Similarly, in 2023, Nguyen Tri Phuong Hospital reported 60.3% multidrug-resistant isolates, with an average ESBL rate of 25.5% [20]. However, comprehensive data on *K. pneumoniae* infections across hospitals remain scare, as resistance reports are typically confined to individual facilities, complication treatment and infection control efforts. This study examines antibiotic resistance in *K. pneumoniae*, focusing on hydrolytic enzyme production including of AmpC β-lactamase, ESBL, and carbapenemase enzyme and resistance genes encoding these enzymes using the MLP-rPCR technique.

## Material and methods

### Study location and collection of clinical samples

**Fig. 1:**
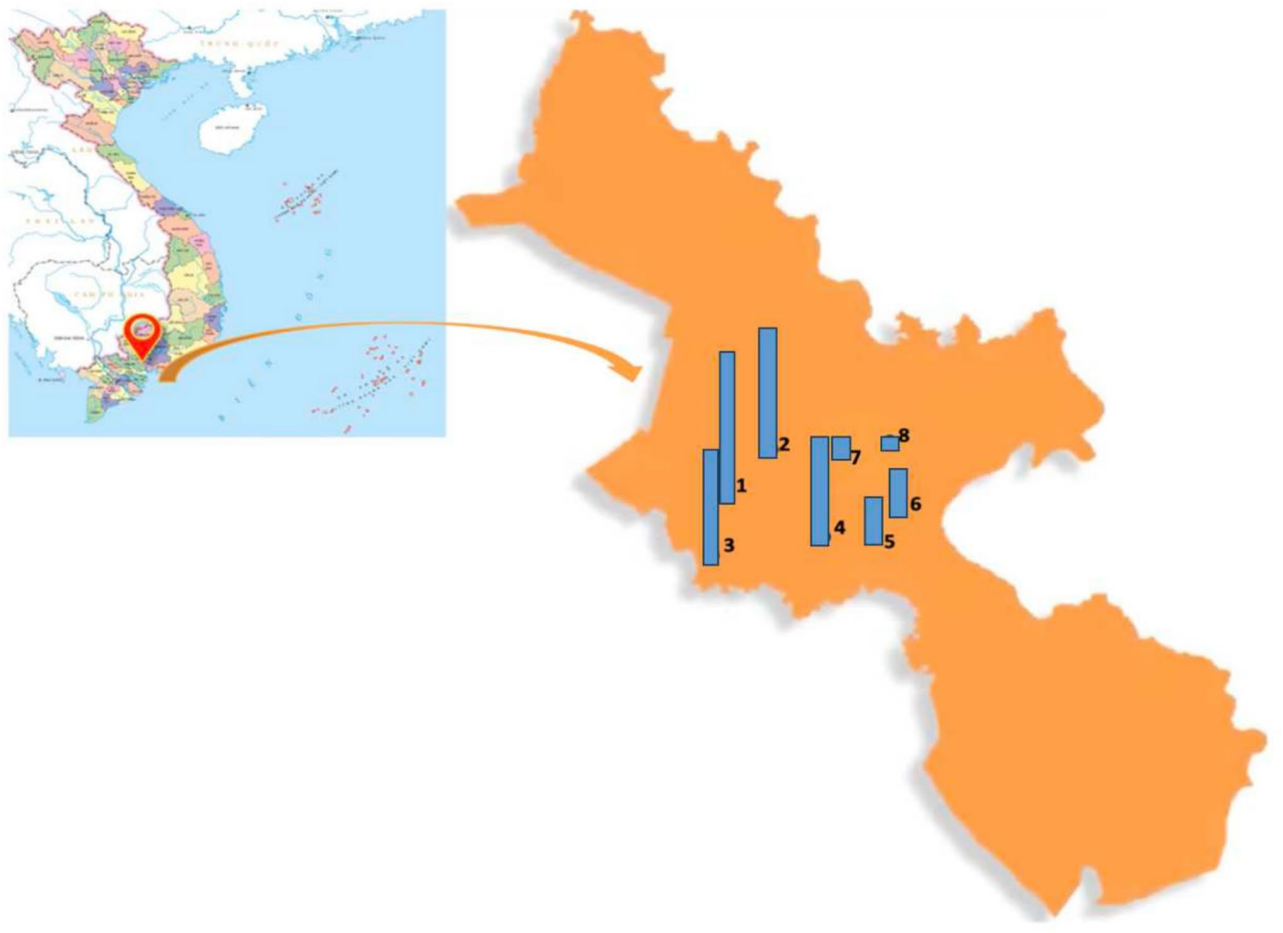
Map of the study area and hospital locations in Ho Chi Minh City, Vietnam for sampling. The blue column represents the number of strains; the taller the column, the greater the number of strains. The exact number of strains is indicated in square brackets. 1/ A Clinic [59], 2/B Hospital [49], 3/C Hospital [48], 4/D Hospital [47], 5/E Hospital [12], 6/F Hospital [12], 7/G Hospital [2], 8/H Hospital [1]

Eight hospitals, including A Clinic, B Hospital, C Hospital, D Hospital, E Hospital, F Hospital, G Hospital and H Hospital, were selected in Ho Chi Minh City, Vietnam. The selection of the eight hospitals was strategically designed to capture the diverse healthcare landscape of Ho Chi Minh City. These hospitals range from small to large facilities, serving as the first point of contact for patients. Additionally, larger tertiary care centers such as hospitals A, B, C and D, which typically manage more complex cases and may encounter higher levels of antibiotic resistance, were included. This range of hospital size was essential for assessing the distribution of resistance across different levels of care. Furthermore, the selected hospitals were geographically distributed across central districts of Ho Chi Minh City (Fig. 1) to ensure a representative sample of the patient population and minimize potential biases related to specific neighborhoods or healthcare accessibility. This study design enhances the generalizability of our findings to the broader context of antimicrobial resistance in Ho Chi Minh City. Located across central districts of Ho Chi Minh City, these hospitals facilitated the collection of a broad and reliable range of clinical samples. The GPS coordinates of these hospitals are provided in Table S1, supplementary.

Clinical samples were collected, from January 2023 to August 2023 and transported to Nam Khoa company’s laboratory as soon as possible, preferably within 2 h after sampling. Requirements for preserving the specimen before and during transportation to Nam Khoa Company’s laboratory are described in Table S2, supplementary. Ensure that specimens are packaged according to the 3-layer principle to effectively preserve samples and ensure biosafety during transportation. Collected specimens are divided into 6 main groups including sputum, pus, urine, bronchial lavage fluid, blood, and other fluids.

### Identification of *K. pneumoniae* by the IDS 14 GNR® biochemical identification

All isolated strains were identified by biochemical identification methods with the IDS 14 GNR® biochemical identification kit [IDS 14 GNR—Nam Khoa Co. Ltd, Vietnam]. Two hundred microliters of bacterial culture, prepared from 2–3 identical colonies in 3 ml of saline at a turbidity of 0.5 McFarland, were dispensed into all wells on the IDS 14 GNR plastic rod. One drop of paraffin was added to wells 1 and 4 before covering the plastic rod. Using an inoculating needle, bacteria were vertically inoculated into the Sulfide Indole Motility medium by inserting the needle one-third of the way to the bottom of the bottle and then removing it vertically. The culture were incubated at 35 ± 2° C for 16–24 h. The biochemical tests on the IDS 14 GNR rod are divided into 5 groups, with groups 1–4 containing three tests each and group 5 containing two tests. After incubation, the total score of each group was calculated, and the results were compared against the IDS 14 GNR identification code system to determine the bacterial species. *K. pneumoniae* was identified with the code 63,361 (Table S3, supplementary). In total, 230 strains of *K. pneumoniae* were collected from clinical samples in Ho Chi Minh City. Only the first strain isolated from each patient was included, and all samples were stored at the laboratory of Nam Khoa Trading and Service Company Limited for further analysis.

### Bacterial study isolates

Two hundred and thirty *K. pneumoniae* clinical isolates obtained in 2023 from these hospitals, in Ho Chi Minh city were used in this study. Quality control in bacterial identification and antibiotic susceptibility testing was performed using *E. coli* ATCC 25922 as a positive control and *P. aeruginosa* ATCC 27853 as a negative control. And the control isolate *P. aeruginosa* A TCC™ 27,853, *K. pneumoniae* ATCC™ 700,603, *K. pneumoniae* ATCC™ BAA 1705 were used in the phenotypic screening of AmpC β-lactamase, ESBL and carbapenemase, respectively. Opposite, *E. coli* ATCC™ 25,922, *K. pneumoniae* ATCC™ BAA 1706 was used as negative control for phenotypic screening of ESBL, AmpC β-lactamase.

### Antimicrobial susceptibility test

The antimicrobial susceptibility of each *K. pneumoniae* isolated was determined by the Kirby-Bauer disk diffusion method using Mueller–Hinton agar at Nam Khoa Laboratories in Vietnam, according to the Clinical and Laboratory Standards Institute guidelines (CLSI) [21]. In brief, bacterial cultures were grown overnight and diluted in sterile Saline to 0.5 McFarland standard and spread, using a cotton-tipped bud across dried MHA-plates. Disks containing ampicilline (AM-10 μg), amoxicillin/clavulanic acid (AMC-30/10 μg), cefoxitin (CFX-30 μg), cefepime (FEF-30 μg), cefotaxime (CTX-30 μg), ceftazidime (CAZ-30 μg), imipenem (IPM-30 μg), were included. The antibiotic disks used were sourced from Nam Khoa Biotek CO., Ltd, Vietnam. Inoculated plates, containing the disk above were incubated aerobically at 37 °C for 18–24 h. Results were interpreted as susceptible, intermediate or resistance, according to the inhibition zone as defined by CLSI, M100-S23 guidelines [22].

### Phenotypic screening of ESBL, AmpC β lactamase and carbapenemase enzymes

**Fig. 2:**
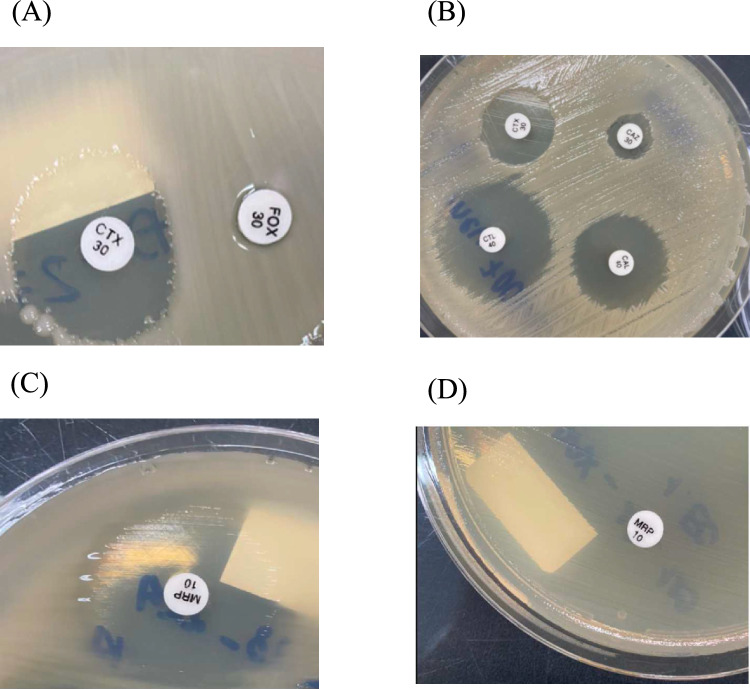
*K. pneumoniae* produces the enzyme iAmpC that forms a D-shape (**A**), produces ESBL enzyme (**B**), do not produce carbapenemase enzymes on mCIM method (**C**) and produce carbapenemase enzymes on mCIM method (**D**)

All isolates included in this study were screened for the production of ESBLs. Susceptibility to cefotaxime (30 μg), cefotaxime/clavulanate (30/10 μg), ceftazidime (30 μg), and ceftazidime/clavulanate (30/10 μg) was tested on Muller Hinton agar. ESBL-producing strains were identified by at increase of at least 5 mm in zone diameter around cefotaxime/clavulanate and ceftazidime/clavulanate disks compared to disks without clavulanic acid (Fig. 2). *E. coli* ATCC 25922 and *K. pneumoniae* ATCC 700603 were used as the control strain [22].

Phenotypic detection of AmpC production was indicated by resistance to a cefoxitin disc (30 μg), as described by Van et al. *Pseudomonas aeruginosa* ATCC 27853 was included as a control strain for each test [23].

**Fig. 3:**
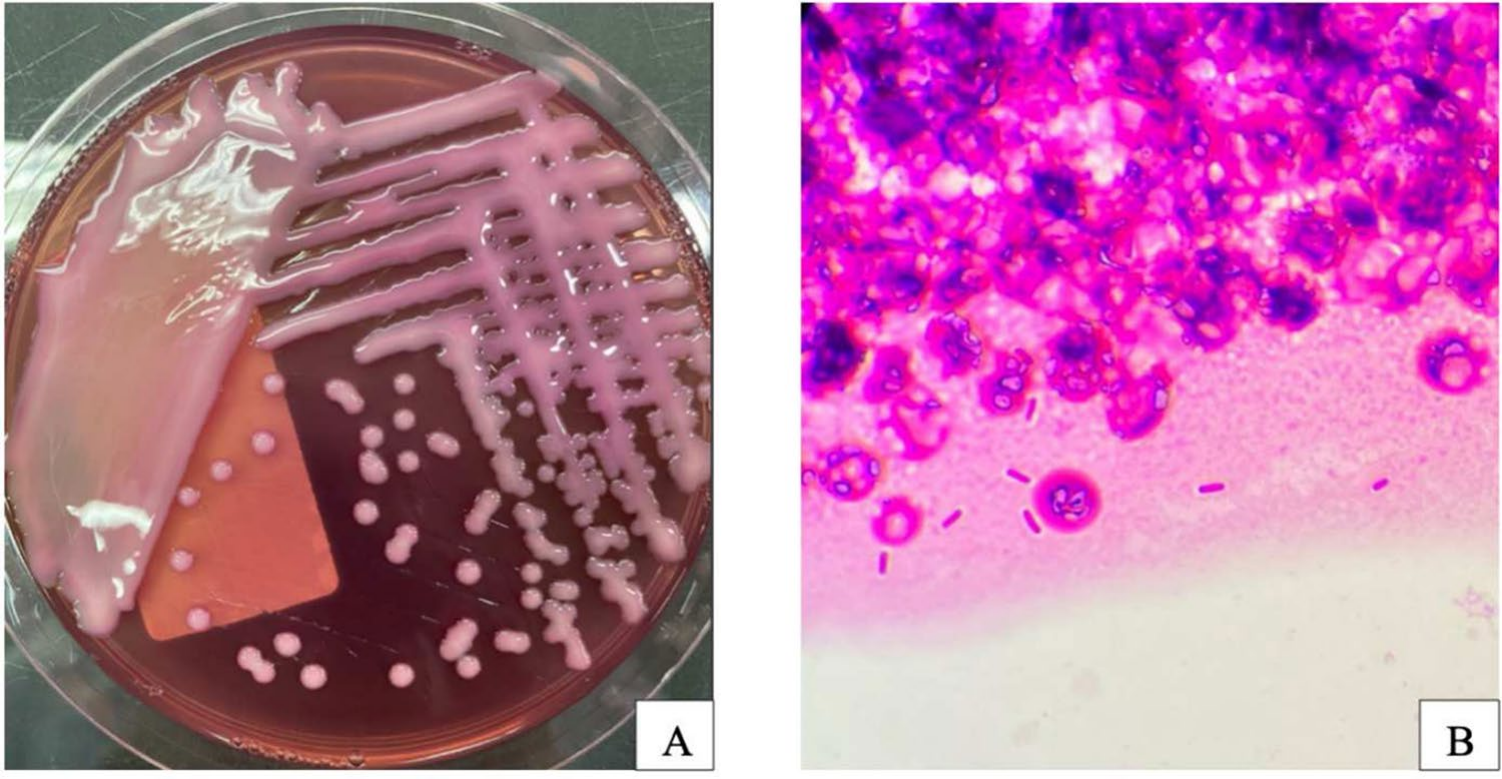
*K. pneumoniae* colonies grown on MC medium (**A**) and bacterial morphology photographed under microscope (**B**)

The carbapenem inactivation method (CIM) using meropenem/imipenem disks was evaluated at 6 h of incubation to confirm inhibition zone formation. The mCIM was conducted following CLSI M100-S33 guidelines [22]. 1 mL of loopful of test isolate was suspended in 2 mL of Tryptone Soy Broth with a meropenem disk, then incubated for 4 h at 35 °C. A bacterial suspension of *E. coli* ATCC 25922, adjusted to McFarland standard n°0.5 was then spread onto Mueller–Hinton agar for routine disk diffusion. The incubated disk was transferred from the Tryptone Soy Broth suspension onto the Mueller–Hinton agar and further incubated for 16 h–20 h at 35 °C. Results were interpreted as positive for inhibition zone diameters of 6–15 mm, negative for ≥ 19 mm and indeterminate for 16–18 mm. *K. pneumoniae* ATCC BAA-1705 and BAA-1706 were used as positive and negative mCIM controls, respectively. All isolates were subsequently subcultured and stored at − 80 °C in 25% glycerol for further molecular study (Fig. 3).

### Detection of ESBL-, AmpC β-lactamase-, and carbapenemase-related genes by Multiplex RT-PCR method

Bacterial DNA was extracted using the Thermo Scientific KingFisher Flex purification system with ^NK^DNARNA prep-MAGBEAD-FLEX extraction chemicals (Thermo Fisher Scientific®). Primers (Table S4, supplementary) were designed by the Vietnam Institute of Clinical Microbiology Research and Development and evaluated for specificity using the BLAST tool from National Center for Biotechnology Information (NCBI) and were manufactured by SFCprobe Company (Korea). Specific primers targeted genes including EBC, DHA, and MOX [24], CIT [25], SHV, TEM, and CMY [26], CTX-M [27], CTX-M1 and CTX-M9 [28], KPC and NDM1 [23], OXA-48 [23, 29]. Multiplex real-time PCR (MPL-rPCR) conditions were: 95 °C for 15 min (hot-start taq polymerase), followed by 40 cycles of 94 °C for 15s and 60 °C for 1 min during fluorescence signals were captured. MPL-rPCR was performed using 5 mix tubes (4 coding genotypes per tube). The reaction volume was 50μL, comprising 40μL of 1.25X Multiplex RT-PCR master mix (1X), 1μL for each primer (10 pmol/μL), 0.5μL of TaqMan probe (10 pm/μL), 0.5μL of purified water, and 5μL of DNA. Results were analyzed using CFX 96 software [Bio-Rad] across color channels HEX, FAM, TexasRED and CY5.

### Sampling size and statistical analysis

The sample size was calculated using the formula for estimating a proportion in a population

$$n\ge {Z}_{1-\frac{\alpha }{2}}^{2}\left(1-p\right)p/{c}^{2}$$

where α = 0.05, p = 0.178 (based on the ESBL-producing *Klebsiella pneumoniae* rate at Thong Nhat Hospital in 2022) [30], and *c* = 0.05 (margin of error). This resulted in a minimum sample size of 224 K*. pneumoniae* isolates, but 230 strains were collected for convenience. All eligible *K. pneumoniae* isolates were included in the analysis. Data were analyzed using EpiData 3.0 software and descriptive statistics (frequencies and percentages) were calculated in Excel to describe antibiotic susceptibility and resistance gene rates.

## Results

### *K. pneumoniae* strains isolation from clinical samples

**Fig. 4:**
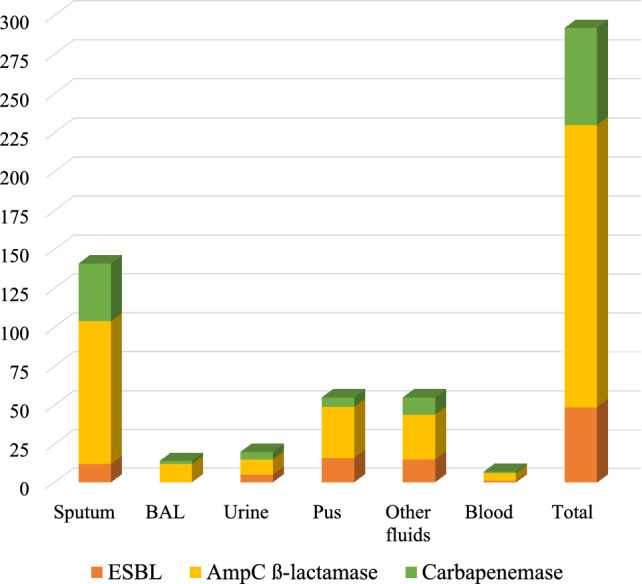
Number of β-lactamase enzymes secreted by *K. pneumoniae* isolated from different specimens, *BAL-Bronchoalveolar Lavage*

A total of 230 K*. pneumoniae* strains meeting the study criteria were isolated and identified from clinical samples, including 104 from sputum, 49 from pus, 43 from other fluids, 15 from urine, 13 from bronchial lavage fluid, and 6 from blood (Fig. 4). *K. pneumoniae* bacteria were most frequently isolated from sputum specimens, while the remaining isolates were obtained from a variety of other specimen types. Morphologically, *K. pneumoniae* colonies cultured on MC medium at 37 °C for 18–24 h present a pink coloration indicative of lactose fermentation. The medium itself experiences no color alteration (Fig S1, supplementary).

### Production of AmpC β-lactamase, ESBL and carbapenemase enzymes in 230 strains of *K. pneumoniae* isolated different clinical samples

The production rates of AmpC β-lactamase, ESBL, and carbapenemase in *K. pneumoniae* were 78.7%, 21.3% and 27%, respectively. Among the 181 K*. pneumoniae* strains producing AmpC β-lactamase, 80.7% were decompressive, while 19.3% were inducible (Table 1).

**Table 1:** Proportion of AmpC β-lactamase, ESBL and carbapenemase production. *iAmpC: inducible AmpC* β*-lactamase; dAmpC: derepressed AmpC* β*-lactamase*

	Enzyme	Total N	Proportion (%)
AmpC β-lactamase	AmpC β-lactamase	230	78.7
*AmpC*	*iAmpC*	181	19.3
	*dAmpC*	181	80.7
ESBL	ESBL	230	21.3
Carbapenemase	Carbapenemase	230	27.0

**Fig. 5:**
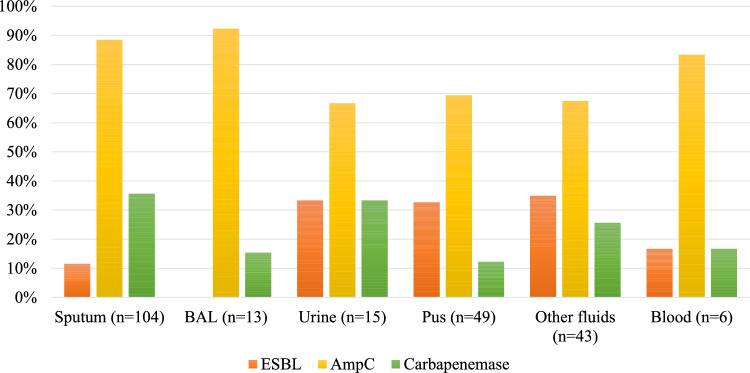
Proportion of β-lactamase enzymes secreted by *K. pneumoniae* isolated from different specimens, *BAL-Bronchoalveolar Lavage*

Among the β-lactamase enzyme production by *K. pneumoniae* from different specimens AmpC β-lactamase production was the most prevalent, ranging from 67.3 to 92.3%; ESBL production ranged from 11.5% to 34.9%; and carbapenemase production ranged from 12.2 to 35.6% (Fig. 5).

Specifically, *K. pneumoniae* strains from bronchial lavage fluid samples showed the highest AmpC β-lactamase production rate at 92.3%; following by sputum (88.5%), blood (83.3%), pus (67.3%), urine (66.7%) and other fluids (67.4%). The highest proportion of ESBL-producing *K. pneumoniae* strains was found in sputum samples at 34.9%, following by urine (33.3%), pus (32.6%), sputum (11.5%), blood (16.7%), and bronchial lavage fluid (12.2%). Carbapenemase-production was most frequently observed in *K. pneumoniae* strains from sputum samples (35.6%), followed by urine (33.3%), other fluid (25.6%), blood (16.7%), bronchial lavage fluid (15.4%), and pus (Fig. 4).

A total of 292 AmpC β-lactamase, ESBL, and carbapenemase enzymes were produced by the 230 K*. pneumoniae* strains. Thus, a significant finding was that 27.0% (62/230) of the *K. pneumoniae* strains exhibited a multi-enzyme phenotype, co-producing various combinations of AmpC β-lactamase, ESBL, and Carbapenemase (Fig. 4).

### Resistant capacity of *K. pneumoniae* strains isolated to ß-lactam antibiotics

**Fig. 6:**
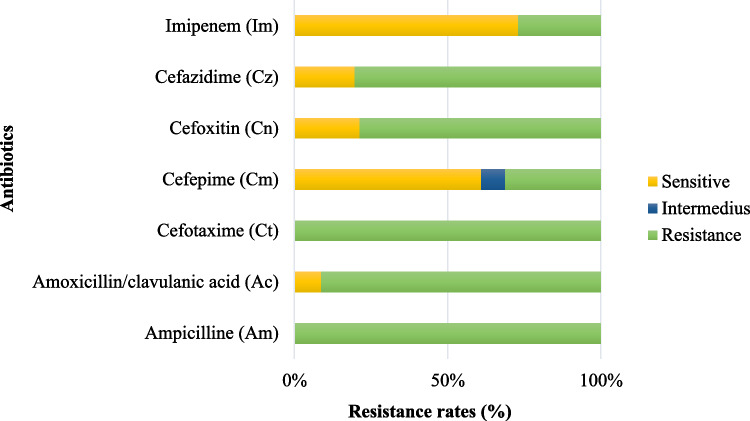
Resistance rates to ß-lactam antibiotics of *K. pneumoniae* strains isolated

*K. pneumoniae* show a high resistant rate of 100% to penicillin group antibiotics, specifically ampicillin. Resistance to ß-lactam/ß-lactamase inhibitor combination antibiotics is also high at 91.3%. Resistant rates within the cephalosporin class range from 31.3–100%, with the highest resistant observed for cefotaxime (100%), cefazidime (80.4%), cefoxitin (78.7%), and cefepime (31.3%). In contrast, resistance to carbapenem antibiotics remains relatively low at 27.0% (Fig. 6).

### Resistance gene rates

The study utilized the MLP-rPCR technique to determine antibiotic resistance genes associated with AmpC β-lactamase, ESBL, and carbapenemase production. Of the *K. pneumoniae* strains studied, the highest prevalence was observed for those carrying genes associated with ESBL, at 90.9% (209/230). Strains carrying genes associated with AmpC β-lactamase and Carbapenemase were found in 67.4% (155/230) and 45.7% (105/230) of the strains, respectively (Table 2).

**Table 2:** Proportion of genes encoding AmpC β-lactamase, ESBL, carbapenemase in isolated *K. pneumoniae* strains

	Total (N)	AmpC β-lactamase encoding gene (N = 230)	ESBL encoding gene (N = 230)	Carbapenemase encoding gene (N = 230)	
Gene-carrying strain	230	67.4%	90.9%	45.7%	

Coding gene		DHA	EBC	CIT	MOX	ACC	SHV	TEM	CTX-M1	CTX-M9	CMY	OXA-48	NDM1	KPC	Total number of genes detected	Gene/strain Ratio
n, %		146 63.5%	10 4.3%	3 1.3%	1 0.4%	0 0.0%	194 84.3%	127 55.2%	115 50%	46 20.0%	2 0.9%	72 31.3%	57 24.8%	21 9.1%		
Sputum	104	76	2	1	0	0	94	62	57	26	1	45	32	8	404	3.9
Pus	49	29	0	2	0	0	39	22	21	9	1	9	8	4	144	2.9
Urine	15	5	2	0	0	0	12	8	9	2	0	5	5	1	49	3.3
BAL	13	12	0	0	0	0	10	6	2	4	0	3	2	3	42	3.2
Blood	16	5	0	0	0	0	6	2	2	1	0	1	1	3	21	3.5
Other fluids	43	19	6	0	1	0	33	27	24	4	0	9	9	2	134	3.1
Total	230	146	10	3	1	0	194	127	115	46	2	72	57	21	794	3.3

*DHA Morganella morganii* AmpC β-lactamase, *EBC Enterobacter cloacae* β-lactamase, *CIT Citrobacter species* β-lactamase, *MOX* Moxalactam hydrolyzing β-lactamase, *ACC* Ambler class C β-lactamase, *SHV* Sulphydryl variable, *TEM* Temoneira, *CTX-M1* Cefotaximase Munich 1, *CTX-M9* Cefotaximase Munich 9, *CMY* Cephamycins hydrolyzing β-lactamase, *OXA-48* Oxacillin Hydrolying Enzyme 48, *NDM1* New Delhi Metallo- β-lactamase 1, *KPC Klebsiella pneumoniae* Carbapenemase

Among the AmpC β-lactamase genes, the DHA genotype was predominant, representing 63.5%, while the MOX, CIT, and EBC genotypes were less common, with frequencies ranging from 0.4% to 4.3%. The ACC genotype was not detected. For ESBL-related genes, the main genotypes identified were mainly SHV, TEM, and CTX-M, with frequencies of 84.3%, 55.2%, and 70.0%, respectively (CTX-M1 at 50.0% and CTX-M9 at 20.0%). The CMY genotype was detected at a low rate of 0.9%. The carbapenemase-related genotypes included OXA-48 at 31.3%, NDM1 at 24.8%, and KPC at 9.1% (Table 2).

Considering strains harboring resistance genes from different clinical sources, the results showed that the 104 strains isolated from sputum samples harbored a total of 404 antibiotic resistance genes, with an average of 3.9 genes per strain. *K. pneumoniae* strains from other sources such as pus, urine, bronchial lavage fluid, blood, and other fluids carried an average of 2.9 to 3.5 genes per strain. Overall, each *K. pneumoniae* strain contained an average of 3.3 antibiotic resistance genes, ranging from 2.9 to 3.9 genes per strain (Table 2).

## Discussion

*K. pneumoniae* was isolated from various clinical specimens, with sputum samples demonstrating the highest prevalence at 45.2%, underscoring its role as a significant contributor to opportunistic and nosocomial infections, particularly respiratory tract infections. Similarly, Nguyen et al. [31] reported a comparable trend, with sputum samples yielding the highest isolation rate at 55.4% [31]. This high detection rate in respiratory secretions reflect the strong association of *K. pneumoniae* with respiratory tract infections, such as pneumonia and bronchitis.

The prevalence of AmpC β-lactamase, ESBL, and carbapenemase production was 78.7%, 21.3%, and 27.0%, respectively, with notable variations compared to finding from other studies. The AmpC β-lactamase production rate (78.7%) was significantly higher than the rates reported at Can Tho General Hospital, where 48.9% was detected using the cefoxitin disc technique and 30.5% using the three-dimensional technique [17]. Similarly, a higher ESBL rate (29.9%) was reported at E Hospital compared to the rate observed in this study [32]. In a broader context, research conducted in 2017 on *K. pneumoniae* causing antibiotic-resistant intra-abdominal infections in the Asia Pacific region reported an ESBL rate of 24.3%, with Thailand exhibiting the highest rate at 36.5% [33]. In Iran, studies showed variable enzyme production rates, with 46.7% for ESBL and 52.8% carbapenemase [34] and 20.0% for AmpC β-lactamase, 40.0% for ESBL, and 43.3% for carbapenemase [35]. The variation in enzyme production rates across studies can be explained by several factors. First, differences in sample sizes, selection criteria, and study designs may influence the results [16]. Second, methodological variations, such as using the cefoxitin disc method *versus* the three-dimensional technique, can yield different outcomes. Some detection techniques may have higher sensitivity or specificity for certain β-lactamase types, leading in discrepancies in reported rates [36, 37]. Cefoxitin resistance can occur not only due to AmpC beta-lactamase production but also through other enzymes, including metallo-beta-lactamase and ESBLs, or non-enzymatic mechanisms such as purine channel mutation. Furthermore, some studies have shown that cefoxitin can be used as a substrate for active efflux pumps in clinical isolates [38]. Regions with high or uncontrolled antibiotics usage often show elevated enzyme production rates due to selective pressure favoring resistant strains. Notably, the overuse of third-generation cephalosporins has been strongly linked to the emergence of ESBL-producing bacteria with high levels of antibiotic resistance [16]. At last, in recent year, *K. pneumoniae* has increasingly producted AmpC β-lactamase and carbapenemase enzymes, correlating with the rising prelavance of cephalosporin-resistant strains.

To gain a deeper understanding of AmpC β-lactamase activity, a focused investigation was conducted to evaluate its specific effectiveness. Among the 181 strains producing AmpC β-lactamase, 80.7% were classified as derepressed AmpC (dAmpC) and 19.3% as inducible AmpC (iAmpC). Initially, bacteria producing iAmpC are sensitive to third-generation cephalosporins; however, the continued use of these antibiotics in treating infectious can lead to mutations. These mutation can result in the bacteria producing high levels of AmpC β-lactamase enzyme, transitioning to dAmpC, and becoming resistance to 3rd generation cephalosporin antibiotics [23, 39]. Consequently, treatment with 3rd generation cephalosporins ultimately becomes ineffective. This mechanism likely explains the high resistance rates to 3rd generation cephalosporin observed in this study, which ranged from 80.4% to 100%. Therefore, early detection of bacteria secreting iAmpC is crucial to prevent treatment failures and mitigate the emergence of resistance to these antibiotics. Moreover, It is interesting to note the co-production of enzyme by individual strains. Strains, coproducing ESBL and high level of AmpC are becoming increasingly common [40]. Among the 230 strains isolated, 27.0% were found to simultaneously produce more than one enzyme type, including combinations of AmpC β-lactamase, ESBL, and carbapenemase. This aligns with findings by Mohanty et al. [41], though our co-production rates were lower than those reported by Handa et al. [42], where 58.4% to 84.6% of *Enterobacterales* strains co-produced AmpC and ESBL enzymes [41, 42]. These results confirm that *K. pneumoniae* can produce multiple enzymes within a single strain, significantly enhancing its resistance to various antibiotic classes and making treatment more challenging. According to the IDSA 2024 guidance on the treatment of antibiotic-resistant Gram-negative infections, consideration should be given if both enzymes can be produced by Enterobacterales, as while cefepime may be effective in treating AmpC-Enterobacterales infections, it remains suboptimal against infections caused by ESBL-producing Enterobacterales [43]. The results of our study realized in 2023 (unpublished) showed the antibiotic resistance rates of *K. pneumoniae* were as follows: 91.3% for amoxicillin/clavulanic acid, 100% for cefotaxime, 80.4% for cefazidime, 78.7% for cefoxitin, 31.3% for cefepime, and 27% for imipenem. In comparaison, a study conducted at E Hospital in Vietnam during 2018–2020 reported *K. pneumoniae* resistance to cephalosporins ranging from 44.7 to 65.7% [32]. By 2022, this resistance rate had increased to 70.6% of third-generation cephalosporins in children with urinary tract infections and urinary tract abnormalities at the National Children’s Hospital, Vietnam [44]. In Iran, the highest resistance rate was observed against amoxicillin 98.2%, followed by cefotaxime 78.2%. The indiscriminate and inappropriate use of antibiotics is one of the main factors contributing to the increase in MDR levels in many areas [38].

Among the 230 K*. pneumoniae* strains isolated and investigated, the prevalence of strains harboring the AmpC β-lactamase gene was 67.4%, significantly higher than the 9.2% detected in a study conducted in Can Tho City, Vietnam [17]. In Iran, the AmpC β-lactamase gene was detected at lower rates compared to our finding, with the rates of 24.4%, 17.5%, and 36%, respectively [35, 45, 46]. Same as the results of AmpC β-lactamase gene, the prevalence of ESBL-related resistance genes in our case was strikingly high at 90.9%, substantially exceeding the rates of 40% and 24% reported by Kazemian et al. (2019) and Owusu et al. (2023), respectively [35, 46]. These results underscore the significant burden of AmpC β-lactamase and ESBL resistance among *K. pneumoniae* isolates from hospitals in Ho Chi Minh City. The universal resistance to third-generation cephalosporins observed in these isolates further supports the hypothesis that they may represent a multidrug-resistant phenotype. Our study revealed a notable prevalence of 45.7% for carbapenemase resistance genes among *K. pneumoniae* isolates. This prelevance is slightly lower than the 46.3% reported by Marwa S. Taha’s study (2023) in Egypt but higher than the 37.8% found in the study by Kazemian’s 2019 [35, 47]. These findings highlight the widespread dissemination of these resistance genes and underscore the urgent need for intensified control measures.

The frequency of genes encoding AmpC β-lactamase, ESBL and carbapenemases are summarized in Table 2. Four of the five AmpC β-lactamase genes were detected at varying proportions, while the ACC gene was not detected. Compared to other studies, variations in gene presence and prevalence were observed. Tran [17] reported 100% of *K. pneumoniae* strains carried the DHA gene at Can Tho General Hospital [17]. Another study realized at the Central Hospital for Tropical Diseases reported a lower prevalence of the DHA (1.8%) and CMY (0.12%) genes [48]. In Iran, Kazemian found 16.6% of strains carrying the DHA gene and 5.5% carrying the CIT gene, whereas Robatjazi et al. [45] observed different rates, with 6.3% for DHA and 9.5% for CIT [35, 45]. Notably, neither study detected the presence of MOX and ACC genes in their isolates. These findings indicate the widespread distribution of the DHA gene, which encodes resistance associated with AmpC β-lactamase. Additionally, the gene encoding AmpC β-lactamase can be located on either the chromosome or plasmid, enabling the widespread dissemination of antibiotic resistance genes, with a high rate of 67.4% [37]. In the case of ESBL-associated genes, Tran (2022) reported that the prevalence of *K. pneumoniae* strains carrying the SHV gene was 98.4%, TEM was 52.2%, and CTX-M was 51.8% [48]. In contrast, Kazemian reported lower rates in Iran, with CTX-M (11.1%), SHV (21.1%), and TEM (23.3%) [35]. In fact, Ambler class A ESBL enzymes are a key contributor to 3rd generation cephalosporin resistance among *Enterobacteriaceae*. Specifically, genetic alterations in broad-spectrum β-lactamases such as TEM-1, TEM-2, and SHV-1 enable them to hydrolyze third- and fourth-generation cephalosporins including cefotaxime, ceftriaxone, and ceftazidime [49]. While the specific distribution of antibiotic resistance genes varies depending on geographical location and study period, SHV, CTX-M, and TEM genes consistently emerge as the predominant genotypes in ESBL-producing strains. Furthermore, our analysis indicates a rising trend in the prevalence of these genes among *K. pneumoniae* isolates from clinical samples, underscoring the growing challenge of managing antibiotic resistance in healthcare settings. For carbapenemase-associated genes, our results are consistent with those of Tran et al. [48], who reported the prevalence of *K. pneumoniae* carrying OXA, KPC, and NDM genes at 46.5%, 45.6%, and 54.5%, respectively [48]. In constrat, Kazemian et al. [35] reported a much lower prevalence, with 14.4% for OXA-48 [35]. According to the report by Nabi Jomehzadeh et al. [34] the OXA-48 type carbapenemase was predominant (19.4%), followed by NDM (8.3%) [34]. Thus, early detection of bacteria carrying carbapenem resistance genes allows clinicians to select appropriate antibiotics such as Ceftazidime-avibactam as the preferred treatment option for OXA-48-producing Enterobacterales infections. Ceftazidime-avibactam in combination with aztreonam, or cefiderocol as monotherapy, are preferred treatment options for NDM and other MBL-producing Enterobacterales infections. Meropenem-vaborbactam, ceftazidime-avibactam, and imipenem-cilastatin-relebactam are preferred treatment options for KPC-producing Enterobacterales infections. Cefiderocol is an alternative [43].

It is interesting to note that the combined analysis of the phenotypic and genotypic results for *K. pneumoniae* strains revealed a significant discrepancy between the two. In fact, while 90.9% of strains carried the ESBL gene, only 21.3% exhibited ESBL enzyme production. This suggests that a substantial proportion of *K. pneumoniae* strains harboring the ESBL gene may go undetected if phenotypic tests are relied upon exclusively. Furthermore, 78.7% of strains produced AmpC enzymes, despite only 67.4% carrying the AmpC gene. This finding indicates that AmpC enzyme activity might mask or inhibit ESBL enzyme activity, leading to false-negative results in phenotypic assays. It can be difficult to accurately detect ESBLs using the disk diffusion method. The identification of AmpC β-lactamase based on cefoxitin resistance can also be misleading due to non-AmpC-related resistance mechanisms, such as efflux pumps or porin changes [43, 50]. Thomson et al. [50] proposed a potential explanation for this phenomenon, noting that confirmatory tests employing ceftazidime and cefotaxime, either alone or in combination with clavulanate, are valuable diagnostic tools but have certain limitations. These tests can generate false-positive results when dealing with KPCs and overproduced K1 β-lactamases, and false-negative results with isolates producing elevated levels of AmpC emphasized the importance of detecting AmpC enzymes to improve the clinical management of infections and provide valuable epidemiological insights [42, 50]. Morever, the CLSI has not established specific guidelines for detecting AmpC-mediated resistance in Gram-negative clinical isolates, often leading to challenges and misleading results, particularly in phenotypic tests. In summary, these findings indicate that phenotypic methods alone may underestimate the prevalence of ESBL-producing strains, as AmpC activity can mask the presence of ESBL genes. This highlights the need for incorporating genotypic testing to achieve accurate detection and effective clinical management.

Research on *Klebsiella pneumoniae* isolates reveals that each bacterial strain carries an average of 3.3 resistance genes, ranging from 2.9 to 3.9 per strain. This indicates a consistently high burden of antibiotic resistance among strains in hospitals across Ho Chi Minh City, regardless of specimen origin. Notably, the widespread presence of β-lactam resistance genes, particularly those conferring resistance to 3rd generation cephalosporins, highlights the similar resistance characteristics among these isolates. These findings provide valuable insights that can aid healthcare professionals in developing more effective treatment regimens for *K. pneumoniae* infections. However, this study was conducted over a short period with a limited sample size, underscoring the need for further research to assess both the phenotypic characteristics and resistance genotypes of *K. pneumoniae* strains from diverse clinical sources and healthcare facilities across different regions. A broader investigation would offer a more comprehensive understanding of resistance profiles and gene distribution in this pathogen. Additionally, the absence of phylogenetic analysis limits the ability to assess genetic relatedness among isolates and hiders understanding of their evolutionary connections. This gap makes it difficult to determine whether the strains share a common origin or are genetically unrelated and dispersed. Future studies should incorporate whole-genome sequencing (WGS) and phylogenetic analysis to elucidate the genetic relationships and transmission mechanisms of drug-resistant *K. pneumoniae* strains. Overall, strengthening infection control measures in both hospitals and community settings is essential to curb the spread of multidrug-resistant and genetically mutated strains. Performing antibiotic susceptibility testing or resistance genes detection prior to prescribing penicillins or 1st to 4th generation cephalosporins is critical for optimizing treatment, particularly given the high resistance of AmpC β-lactamase and extended-spectrum β-lactamase (ESBL) producers.

## Conclusion

This study provides a concerning depiction of the complex antibiotic resistance landscape of *K. pneumoniae* in Ho Chi Minh City hospitals, highlighting the diversity and severity of resistance mechanisms observed. The widespread presence of strains carrying multiple resistance enzymes concurrently, particularly AmpC β-lactamases, ESBLs, and carbapenemases, poses a significant challenge to effective treatment. The diversity of resistance genes detected, notably SHV, TEM, CTX-M (with CTX-M1 predominating) for ESBLs and OXA-48, NDM1, KPC for carbapenemases, further complicates the antibiotic resistance picture. These findings have critical implications for the development and adaptation of infection control policies and antibiotic stewardship programs. The high rates of resistance to 3rd generation cephalosporins, driven by these diverse resistance mechanisms, underscore the need for reassessing current treatment protocols. This data provides a robust scientific basis for developing local empirical treatment guidelines for *K. pneumoniae* infections, aiming to optimize antibiotic use and preserve the efficacy of last-resort agents. Simultaneously, the study emphasizes the importance of strengthening infection prevention and control measures to curb the spread of these highly resistant strains within hospital settings. Continuous surveillance of resistance trends and molecular characterization of circulating strains are essential to guide ongoing efforts in the fight against antibiotic resistance.

## Supplementary Information

Below is the link to the electronic supplementary material.

Supplementary file1 (DOCX 847 KB)

## Data Availability

No datasets were generated or analysed during the current study.
