# Supplementary material for: Characterization of phenotypic and genotypic traits of Klebsiella pneumoniae strains resistant to 3rd generation cephalosporins in hospital settings: A case study in Ho Chi Minh City, Vietnam
Source: Mol Biol Rep. 2025 Apr 15;52(1):396. doi: 10.1007/s11033-025-10493-4 (PMC12000183; doi:10.1007/s11033-025-10493-4)
Supplement: Supplementary file 1 — Supplementary file1 (DOCX 847 KB) [file 11033_2025_10493_MOESM1_ESM.docx]

**Characterization of phenotypic and genotypic traits of *Klebsiella pneumoniae* strains resistant to 3^rd^ generation cephalosporins in hospital settings: A case study in Ho Chi Minh City, Vietnam**

Minh Tuan HUYNH^1,2^, My Dung JUSSELME^3*^, Hung Van PHAM4, Tien Dung NGUYEN5, Minh Quynh CHAU^[[1]](#footnote-1)**^

^1^Medical Microbiology Department, University Medical Center Ho Chi Minh City, 215 Hong Bang street, Ward 11, District 5, Ho Chi Minh City, Vietnam

^2^Department of Microbiology and Parasitology, University of Medicine and Pharmacy at Ho Chi Minh City, 217 Hong Bang street, Ward 11, District 5, Ho Chi Minh City, Vietnam

3LEESU, Univ Paris Est Creteil, ENPC, Institut Polytechnique de Paris, Creteil, France

^4^Nam Khoa Trading and Service Company Limited, 793/58 Tran Xuan Soan street, Tan Hung Ward, District 7, Ho Chi Minh City, Vietnam

5National Fisheries Inspection and Quanlity Assurance Branch 4, 271 To Ngoc Van street, Linh Dong Ward, Thu Duc City, Ho Chi Minh City, Vietnam

**Table S1.** GPS latitude and longitude coordinates of sampling sites (Hospital in Ho Chi Minh city)

| **Sampling site** | **Address** | **Latitude** | **Longitude** |
| --- | --- | --- | --- |
| A Clinic | Lanh Binh Thang street, District 11, Ho Chi Minh City | 10°45'44.7"N | 106°39'21.7"E |
| B Hospital | Le Hong Phong street, District 10, Ho Chi Minh City | 10°46'18.4"N | 106°40'15.4"E |
| C Hospital | Binh Tri Dong B street, Binh Tan District, Ho Chi Minh City | 10°44'25.9"N | 106°36'25.9"E |
| D Hospital | Nguyen Trai street, District 5, Ho Chi Minh City | 10°45′19″N | 106°40′13″E |
| E Hospital | An Binh street, District 5, Ho Chi Minh City | 10°45'14"N | 106°40'19"E |
| F Hospital | Nguyen Trai street, District 5, Ho Chi Minh City | 10°45'23.2"N | 106°40'30"E |
| G Hospital | Su Van Hanh street, District 10, Ho Chi Minh City | 10°46′09″N | 106°40′15″E |
| H Hospital | Ly Tu Trong street, District 1, Ho Chi Minh City | 10°46′51″N | 106°42′09″E |

**Table S2.** Requirements for preservation of specimens before and during transport.

| **Specimen** | **Storage conditions** | **Storage time** |
| --- | --- | --- |
| Sputum | 18 – 30^0^C | < 24h |
| Pus | 18 – 30^0^C | < 24h |
| Urine | 4 – 8^0^C | < 24h, Do not store samples at 0^0^C |
| Blood | 18 – 30^0^C | < 24h |
|  | 4 – 8^0^C | ≥ 24h and < 48h |
| Bronchial lavage fluid | 18 – 30^0^C | < 24h |
| Cerebrospinal fluid Other fluids | 4 – 8^0^C  18 – 30^0^C | ≥ 24h and < 48h  < 24h |

**Table S3.** Biochemical results for the identification of *K. pneumoniae* bacteria using IDS14 GNR. Identification code: 63361. (1) GLU: Glucose positive, yellow solution. (2) NIT: Nitrate reduction to nitrite is indicated by a red or pink color in the solution within 5 minutes. (3) ONPG: Ortho-Nitrophenyl-beta-galactoside, ONPG positive when the solution is yellow or pale yellow. (4) URE: Urea-producing bacteria are indicated by a rose-pink or light red solution. (5) PDA: Phenylalanine deaminase, After adding 1 drop of FeCl3, a PAD positive result is indicated by a green solution, which may fade over time. (6) CIT: Citrate utilization by bacteria is indicated by a blue or light blue solution. (7) ESC: Esculin hydrolysis by bacteria is indicated by a black solution. (8) H2S: H2S-producing bacteria are indicated by a black precipitate at the bottom of the tube. (9) IND: Indol, After adding Kovac's reagent, an indole-producing bacteria is indicated by a red ring on the surface of the solution. Read the results immediately, within 5 minutes. (10) VP: Voges-Proskauer, After adding KOH and alpha-naphthol solutions, a VP positive result is indicated by a red solution. This appears after 5 minutes, but no later than 2 hours. (11) MLO: Malonate, Malonate utilization by bacteria is indicated by a blue or light blue solution. (12) LDC: Lysin decarboxylase, LDC-producing bacteria are indicated by a purple solution. (13) SIM: Sulfide Indole Motility, SIM positive: The medium turns red, spreading outwards from the inoculation line, potentially spreading to the surface of the medium (aerobic bacteria) or forming a hazy area around the inoculation line.

Biochemical test results: Glucose fermentation positive, nitrate reduction to nitrite positive, ONPG positive, urea positive, PAD negative, CIT positive, ESC positive, H_2_S negative, IND negative, VP positive, MLO positive, LDC positive, motility negative.

| **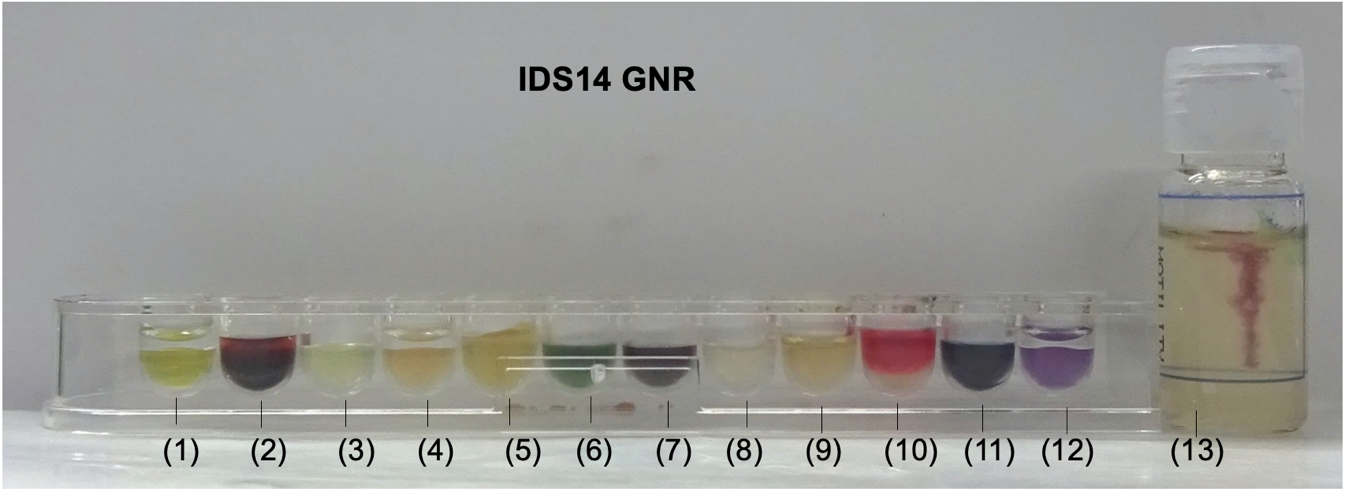** |
| --- |

**Table S4.** Primer sequences and taqman probes performed in the study. DHA- *Morganella morganii* AmpC β-lactamase, EBC- *Enterobacter cloacae* β-lactamase, CIT- *Citrobacter species* β-lactamase, MOX- Moxalactam hydrolyzing β-lactamase, ACC - Ambler class C β-lactamase, SHV- Sulphydryl variable, TEM- Temoneira, CTX-M1 - Cefotaximase Munich 1, CTX-M9 - Cefotaximase Munich 9, CMY- Cephamycins hydrolyzing β-lactamase, OXA-48 - Oxacillin Hydrolying Enzyme 48, NDM1- New Delhi Metallo- β-lactamase 1, KPC- *Klebsiella pneumoniae* Carbapenemase. F- Forward, R- Reverse, P- Primer.

| **Gene** | **Primer** | **Sequences 5’🡪 3’** | **Reference** |
| --- | --- | --- | --- |
| EBC | EBC-F | CAGGCCATTCCGGGTATGG | [24] |
|  | EBC-R | CTTCCACTGCGGCTGCCAGTT |  |
|  | EBC-P | HEX-TAGAGCCCAGCTCAAACAG-BHQ |  |
|  |  |  |  |
| DHA | DHA-F | TTATCTCACACCTTTATTACTG | [24] |
|  | DHA-R | TATCTTTTGAGGCGGATT |  |
|  | DHA-P | FAM-CCGTAAGATTCCGCTCAAGC-BHQ |  |
|  |  |  |  |
| MOX | MOX-F | AGACCCTGTTCGAGATAG | [24] |
|  | MOX-R | ATGGTGATGCTGTCAAAG |  |
|  | MOX-P | CY5-CGTGAGCAAGACCCTGACTG-BHQ |  |
|  |  |  |  |
| CIT | CIT-F | TGGCCAGAACTGACAGGC | [25] |
|  | CIT-R | TTTCTCCTGAACGTGGCTGGC |  |
|  | CIT-P | FAM-GCTTTATCCCTAACGTCATCGGG-BHQ |  |
|  |  |  |  |
| SHV | SHV-F | TCCCATGATGAGCACCTTTAAA | [26] |
|  | SHV-R | TCCTGCTGGCGATAGTGGAT |  |
|  | SHV-P | HEX-TGCCGGTGACGAACAGCTGGAG-BBQ |  |
|  |  |  |  |
| TEM | TEM-F | GCATCTTACGGATGGCATGA | [26] |
|  | TEM-R | GTCCTCCGATCGTTGTCAGAA |  |
|  | TEM-P | FAM-CAGTGCTGCCATAACCATGAGTGA-BHQ |  |
|  |  |  |  |
| CMY | CMY-F | GGCAAACAGTGGCAGGGTAT | [26] |
|  | CMY-R | AATGCGGCTTTATCCCTAACG |  |
|  | CMY-P | CY5-CCTACCGCTGCAGATCCCCGATG-BHQ |  |
|  |  |  |  |
| CTX-M | CTX-M-F | AATCTGACGCTGGGTAAAG | [27] |
|  | CTX-M-R | CCGCTGCCGGTTTTATC |  |
|  | CTX-M1-P | TexasRED-CCCGACAGCTGGGAGACGAAACGT-BHQ | [28] |
|  | CTX-M9-P | TexasRED-CTGGATCGCACTGAACCTACGCTGA-BHQ |  |
|  | CTX-M^*^ | CY5-CGACAATACTGCCATGAA-MGB-BHQ |  |
|  |  |  |  |
| KPC | KPC-TQF | GGCCGCCGTGCAAT AC | [23] |
|  | KPC-TQR | GCCGCCCAACTCCTTCA |  |
|  | KPC-TQP | FAM-TGATAACGCCGCCGCCAATTTGT-BHQ1 |  |
|  |  |  |  |
| NDM1 | NDM1-TQF | GACCGCCCAGATCCTCAA | [23] |
|  | NDM1-TQR | CGCGACCGGCAGGTT |  |
|  | NDM1-TQP | HEX-TGGATCAAGCAGGAGAT-BHQ1 |  |
|  |  |  |  |
| OXA-48 | OXA-48-F | AACGGGCGAACCAAGCA | [29] |
|  | OXA-48-R | TCCTTAACCACGCCCAAATC |  |
|  | OXA-48-P | CY5-CCTTTAAAATTCCCAATAGCTTGATCGCCC-BHQ |  |

****CTX-M probes of all groups except CTX-M1***

|  |
| --- |

1. Corresponding authors:

   *Email: [jusselme@u-pec.fr](mailto:jusselme@u-pec.fr)

   Full postal address :

   Laboratory of Water, Environment and Urban Systems (Leesu)

   Univ Paris Est Creteil, ENPC, Institut Polytechnique de Paris

   61 avenue du Général de Gaulle, 94000-Créteil, France

   **Email: [quynh.cm@umc.edu.vn](mailto:huynhtuan@ump.edu.vn)

   Full postal address:

   Medical Microbiology Department

   University Medical Center Ho Chi Minh City

   215 Hong Bang street, Ward 11, District 5, Ho Chi Minh City, Vietnam [↑](#footnote-ref-1)
